# Diagnostic significance of cerebrospinal fluid flow cytometry in Chinese children with B lineage acute lymphoblastic leukemia

**DOI:** 10.1186/s12887-024-04684-4

**Published:** 2024-03-22

**Authors:** Xue Tang, Huirong Mai, Lulu Wang, Shiyang Chen, Fen Chen, Tonghui Li, Yi Liu, Guichi Zhou, Shilin Liu, Ying Wang, Sixi Liu, Xiaoying Fu, Feiqiu Wen

**Affiliations:** 1 Department of Hematology and Oncology, Shenzhen Children’s Hospital, No.7019 Yitian Road, Futian District, Shenzhen, China; 2 Department of Hematology and Oncology, Shenzhen Children’s Hospital of China Medical University, Shenzhen, China; 3 Department of Laboratory Medicine, Shenzhen Children’s Hospital, Shenzhen, China

**Keywords:** Central nervous system, Cytomorphology, Flow cytometry, Acute lymphoblastic leukemia

## Abstract

**Background:**

Central nervous system leukemia (CNSL) is one of the major causes of the poor prognosis of childhood leukemia. We aimed to compare the sensitivity of cytomorphology (CM) and flow cytometry (FCM) in diagnosing CNSL, emphasizing the importance of FCM in the diagnosis process.

**Methods:**

One-hundred-sixty-five children with newly diagnosed B-cell Acute Lymphoblastic Leukemia (B-cell ALL) were included in this study. Cerebrospinal fluid (CSF) samples were taken for routine CSF analysis, CM analysis, and FCM examination. Computed tomography scans and/or magnetic resonance imaging were performed at diagnosis. Patients with CNS2, CNS3, and traumatic lumbar puncture (TLP) at diagnosis received two additional courses of triple intrathecal injections during induction treatment. We compared the sensitivity of FCM and CM in the diagnosis of children with CNSL.

**Results:**

One hundred and twenty-eight (77.58%) CSF samples were negative by either CM or FCM (CM^−^/FCM^−^), four (2.42%) were positive by both CM and FCM (CM^+^/FCM^+^), and thirty-three (20%) displayed a single positive finding by FCM (CM^−^/FCM^+^) (*p* = 0.044). By adding two intrathecal injections in the induction treatment, ten children with TLP^+^ had no CNS relapse, like those with TLP^−^. However, compared to CNS1 and TLP, the event-free survival (EFS) did not significantly improve in patients with CNS2 and CNS3. Moreover, CNSL status was associated with worse 3-year EFS (*p* < 0.05).

**Conclusions:**

We have validated that FCM is more accurate in stratifying the status of the CNS compared to CM analysis. However, to improve the EFS rate of childhood leukemia, it is necessary to combine CM examination, FCM, and cranial imaging for the early diagnosis of CNSL.

## Background

Central nervous system leukemia (CNSL) is extramedullary leukemia caused by the infiltration of leukemia cells into the meninges, cranial nerves, brain tissue, or spinal cord, which is common in patients with acute leukemia, especially in those with acute lymphoblastic leukemia (ALL) [1]. With the development of chemotherapy and bone marrow transplantation, the complete remission (CR) rate and 5-year event-free survival (EFS) rate of childhood ALL reach more than 90% [2]. Nevertheless, CNSL is still one of the most important causes of the poor prognosis of childhood leukemia, with the central nervous system (CNS) being a predominant extramedullary recurrence site. Indeed, CNS involvement occurs in 3–5% of newly diagnosed acute leukemia and 30–40% of relapsed acute leukemia [3], representing one of the leading causes of death in children with leukemia.

Routine cytological examination of CSF is the gold standard for diagnosing CNS involvement in ALL. However, previous studies demonstrated that using CM as a diagnostic criterion for CNSL might underestimate the morbidity of children with newly diagnosed ALL [4]. Increasing the accuracy of early diagnosis of CNSL and applying appropriate treatment in time greatly improve the EFS rate of childhood leukemia and the quality of life [5, 6].

In this study, we retrospectively analyzed the CSF of 165 children with newly diagnosed acute B-lineage lymphoblastic leukemia. We compared the sensitivity of FCM and CM methods in diagnosis of CNSL, and determined the impact of CNSL at diagnosis on the EFS of childhood B-cell ALL in our center.

## Methods

### Patients’ information

The biological and clinical characteristics of the patients are summarized in Table 1. From November 2019 to May 2021, 165 children with newly diagnosed B-cell ALL were hospitalized in the Department of Hematology and Oncology of Shenzhen Children’s Hospital. The diagnosis met the 2016 WHO diagnostic criteria for leukemia [7], and the CSF of the children came from the first lumbar puncture at the time of treatment. In accordance with the CCCG-ALL-2015 protocol [8], CNSL prophylaxis was administered through intrathecal administration of dexamethasone, methotrexate, and cytarabine. The dosages were tailored based on the patient’s age, with a weekly application for two times for the low-risk group and three times for the intermediate and high-risk group during induction therapy. Additionally, two supplementary lumbar punctures were performed on D9 and D15 if the initial lumbar puncture resulted in injury or the first CSF examination indicated CNS2 or CNS3/CNSL status. Cranial irradiation was omitted for all patients.

**Table 1:** Characteristics of ALL patients (*n* = 165)

Gender, M/F (*n*)	89/76
Median age, year (range)	4y1m (2 m-16y6m)
Molecular (*n*, %)	
*TEL::AML1*	35 (21.21%)
*TCF3::PBX1*	10 (6.06%)
*BCR::ABL1*	5 (3.03%)
*MLL-r*	10 (6.06%)
Normal	85 (51.52%)
Others	20 (12.12%)
WBCc×10^9^/L at diagnosis (*n*, %)	
0–10	89 (53.94%)
10–50	46 (27.88%)
50–100	16 (9.70%)
> 100	14 (8.48%)
Traumatic lumbar puncture	28 (16.97%)
Follow up time, year (range)	2.07 (1.17-3)

WBCc: White blood cell count

### Sample Collection

To ensure the accuracy of results and prognosis, experienced doctors performed the first lumbar puncture of all children under general anesthesia and aimed for a successful puncture on the first attempt. Two mL of CSF were collected from each tube, placed in a sterile container, and immediately sent for examination. Finally, a CSF routine, cell morphology, and FCM examination were carried out.

### Flow cytometry

1 ∼ 2 mL CSF was collected in a sterile tube within 6 h of collection. To prepare the sample, CSF was washed once, centrifuged at 1500 rpm for 5 min. A seven-color FCM combination (anti-human-CD20-FITC,anti-human-CD19-APC,anti-human-CD10-PE-Cy7,anti-human-CD3-APC-Cy7,anti-human CD45-V500, anti-human-CD34 -PerCP-cy5.5 and selected one or two markers among CD58/66c/123/33 PE based on abnormal phenotype of tumour cells in the bone marrow at the initial diagnosis of leukemia) were used to label the cell populations (Table 2). Antibodies and specimen were mixed and incubate for 15 min at room temperature away from light. Followed by washing twice with 2 mL of PBS. Samples were analyzed on a FACSCanto II cytometer (BD Biosciences, San Jose, CA, USA), all antibodies were sourced from Becton Dickinson (Mountain View, CA, USA) and Beckman Coulter (Brea, CA, USA), and the analysis was performed using Diva software 7.0.

**Fig. 1:**
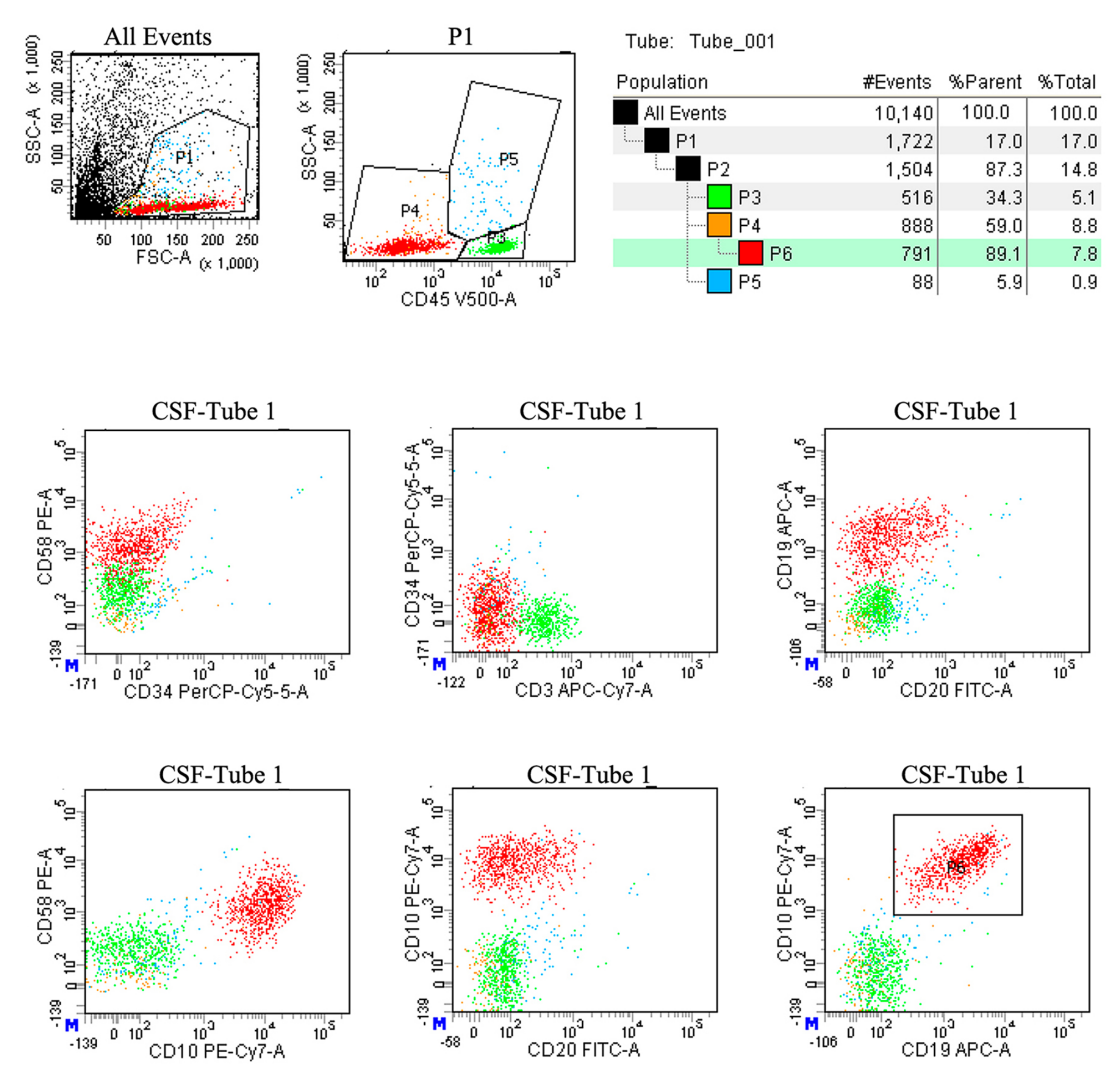
Representative flow cytometry result of CSF in children with B-ALL. Positive report diagnostic results: 52.5% of nucleated cells occupied by abnormal cells; Expressed: CD19, CD20, CD58; Partially expressed: CD34, CD45; Not expressed: CD3, CD20; Conclusion: 1504 nucleated cells were detected in the cerebrospinal fluid, including 516 cells in the mature lymphocyte population (green color cell cluster), 88 cells in the granulocyte population (blue color cell cluster), 97 cells in the nucleated/mature red cell population (orange color cell cluster) and 791 cells in the abnormal B lymphocyte population (red color cell cluster). The percentage of nucleated cells is 52.5%. The immunophenotype is abnormal

The CSF specimen has a small number of cells, a prepared test template has been established and the sample cells are collected at the same time after loading the sample. It is extremely important to keep the injection needle and tube clean to prevent contamination between specimens. Each tube of specimen needs to be rinsed with distilled water prior to sampling until the distilled water is free of particles before the specimen can be tested. Due to the low number of cells in the CSF, cells should be obtained immediately from the time of loading until all cells have been obtained, but do not walk away empty. Generally, 500 ∼ 10,000 cells can be collected, cell counting were by manual (chamber) counting. General gating strategy: FSC-A and SSC-A set up live cell gates to exclude dead cells and debris background noise, CD45/SSC gates were required to set up normal background cell populations, starting with a single live cell gate for analysis. The cellular composition of the CSF was relatively simple. In general, the only two populations of normal CSF were mature lymphocytes and monocytes, with T cells predominating among the lymphocytes. In contrast to bone marrow, the CSF cell count was less, therefore the report would provide absolute counts in addition to percentages. A sample was classified as FCM + when a cluster of at least 25 events with neoplastic features was detected. Clusters of 10 to 24 events with neoplastic features were classified as suspicious. Clusters with less than 10 events were classified as negative (FCM-). Representative flow cytometry result of CSF in children with B-ALL was shown in Fig. 1.

**Table 2:** The Template of CSF flow assay for B-ALL neoplastic cells

Fluorescent	FITC	PE	PerCP-cy5.5	APC-Cy7	APC	PeCy7	V500
CD marker	CD20	CD58/66c/33/123 (select one or two markers based on abnormal phenotype of neoplastic cells in the bone marrow at the initial diagnosis of leukemia)	CD34	CD3	CD19	CD10	CD45

### Cytomorphology

1 ∼ 2 mL of CSF, centrifuged within 1 h of collection, discarded the supernatant, morphologic examination of cells was performed by cytospin preparation stained with Wright-Giemsa. Results were interpreted by experienced hematology cytologists and reported as positive (i.e., presence of tumor cells), negative (i.e., absence of tumor cells), or suspicious when a few atypical cells were detectable.

### CNS leukemia classification

According to CCCG-ALL-2015 chemotherapy protocol [8] shown in Table 3, CNS status was classified as CNS1, CNS2, CNS3 (referred to CNSL), and traumatic lumbar puncture.

**Table 3:** Diagnostic criteria for central nervous system leukemia according to CCCG-ALL-2015

CNS status	Diagnostic criteria
CNS1	CSF detection showed no leukemic cells and no abnormal clinical and imaging changes.
CNS2	a. Cerebrospinal fluid leukocytes < 5 cells/µl, blasts identified through CM in the centrifuged specimen; b. Flow cytometry detected immature cells in the cerebrospinal fluid.
CNS3/CNSL	a. Cerebrospinal fluid leukocytes ≥ 5 cells/µL and blasts were found through CM in the centrifuged specimen; b. Have definite symptoms and signs of central nervous system involvement, such as cranial nerve paralysis that other causes cannot explain; c. Imaging evidence of central nervous system infiltration.
TLP	Bloody cerebrospinal fluid or cerebrospinal fluid red blood cells ≥ 10/µL.

CNS: Central nervous system; TLP: Traumatic lumbar puncture

### Data analysis

In cases with small numbers, chi-squared analysis and Fisher’s exact test were used to compare categorical variables. The non-parametric Mann–Whitney U-test was applied to continuous variables. EFS was calculated from the time between diagnosis and the first event, including relapse, death of any cause, or the point of the last follow-up. Differences with *P* < 0.05 were considered statistically significant.

## Results

### Patients’ characteristics

Overall, among 165 patients with childhood B-cell ALL, 128 CSF samples (77.58%) were negative by both CM and FCM (CM^−^/FCM^−^), 4 CSF samples (2.42%) were positive by both CM and FCM (CM^+^/FCM^+^), and 33 CSF samples (20.0%) displayed a single positive by FCM (CM^−^/FCM^+^) (*p* = 0.044) (Tables 4 and 5). Computed tomography scans and/or magnetic resonance imaging were performed at diagnosis in all patients. Interestingly, in two patients, evidence of leukemic infiltration into the brain parenchyma was discovered by imaging, while both CSF CM and FCM were negative. Additionally, no significant differences were found in the frequencies of fusion genes, including *BCR::ABL1*, *TCF3::PBX1*, *MLL-r*, and *TEL::AML1*, between patients with different CNS statuses (Table 6).

**Table 4:** FCΜ versus CM for detection of CSF tumor cells

	FCM ^+^	FCM ^−^	Total
CM^+^	4(2.42%)	0	4(2.42%)
CM^−^	33(20.0%)	128(77.58%)	161(97.58%)
Total	37(21.21%)	128(78.79%)	165 (100%)

FCΜ: Flow cytometry; CM: Cytomorphology

**Table 5:** The correlation between CSF CM and FCM results and the CSF status

CNS status	CNS1	CNS2	CNS3	TLP	Total
CM^−^/FCM^−^	108	0	2	18	138
CM^−^/FCM^+^	0	23	0	10	33
CM^+^/FCM^−^	0	0	0	0	0
CM^+^/FCM^+^	0	0	4	0	4
Total	108	23	6	28	165

**Table 6:** Characteristics of childhood B-cell ALL in different CNS statuses at diagnosis

Characteristics		Total	CNS1		CNS2		CNS3		TLP+		TLP-		*P*-value
		165	*n*	**%**	*n*	**%**	*n*	**%**	*n*	**%**	*n*	**%**	
Gender	Male/Female	89 (53.94%)	72/36	67/33	8 /15	35/65	3 /3	50/50	6 /4	60/40	6 /12	33/67	0.011
Age(< 10/≥10 years)		149/16	99/9	92/8	22/1	96/4	6/0	100/0	9/1	90/10	13/5	72/28	0.1188
WBC at diagnosis(< 100/≥100 × 10^9^/L)		151/14	99/9	92/8	20/3	87/13	5/1	83/17	9/1	90/10	18/0	100/0	0.3980
WBC at LP(< 5/≥5 × 10^9^/L)		138/27	90/18	83/17	16/7	70/30	5/1	83/17	10/0	100/0	17/1	94/6	0.1569
Risk group	LR/IR/HR	72/91/2	51/55/2	71/60/100	13/10/0	18/11/0	0/6/0	0/7/0	2/8/0	3/9/0	6/12/0	8/13/0	0.1252
*BCR::ABL1*	(Negative/Positive)	160/5	105/3	97/3	22 /1	96/4	5 /1	83/17	10/0	100/0	18/0	100/0	0.303
*TCF3::PBX1*	(Negative/Positive)	155/10	101/7	94/6	23 /0	100/0	6/0	100/0	10/0	100/0	15/3	83/17	0.257
MLL-r	(Negative/Positive)	155/10	102/6	94/6	21/2	91/9	5 /1	83/17	10/0	100/0	17/1	94/6	0.563
*TEL::AML*	(Negative/Positive)	131 /34	85/23	79/21	19/4	83/17	6/0	100/0	8 /2	80/20	13/5	72/28	0.77
Relapse	No/Yes	157/8	106/2	98/2	19/4	83/17	4/2	67/33	10/0	100/0	18/0	100/0	0.002

ALL: acute lymphoblastic leukemia; CNS: central nervous system; TLP+: traumatic lumbar puncture positive; TLP-: traumatic lumbar puncture negative; WBC: white blood cell; LP: lumbar puncture

### Clinical outcomes

**Fig. 2:**
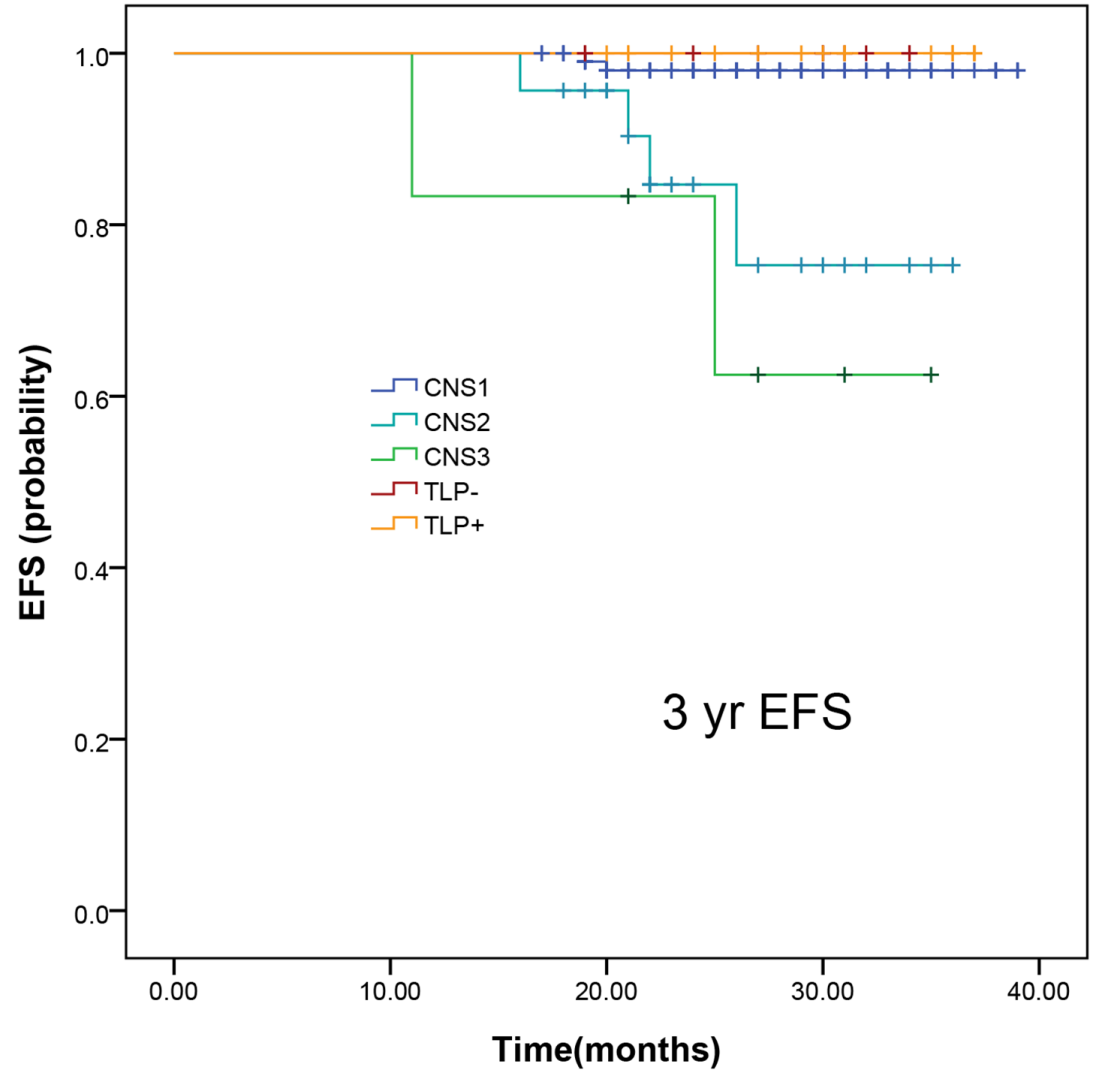
Survival curves of childhood B-cell ALL patients according to CNS status

Of the 165 patients, recurrence occurred in 8 (4.85%) in CNS1, CNS2 and CNS3 statuses but none in TLP statuses (**Table 6**). Among the 8 recurrent B-ALL patients, 3 experienced a relapse of CNSL, 2 had BM relapse, 2 had a combined relapse involving both BM and the CNS, and 1 had a relapse involving both BM and testicles. Of the 8 patients who relapsed, half of them had a *BCR::ABL1* or *TCF3 ::PBX1* fusion. The EFS rate for CNS1, CNS2, CNS3, TLP^+^, and TLP^−^ is shown in Fig. 2 (*p* < 0.05). There was no CNS relapse among ten patients with TLP^+^. However, there was no significant improvement of event-free survival (EFS) in patients with CNS2 and CNS3 compared to those with CNS1 and TLP. Additionally, a worse 3-year EFS (*p* < 0.05) was observed in patients with CNSL status.

We evaluated the survival data for all patients. As shown in Fig. 2, patients with CNS2 and CNS3 at diagnosis had a significant worse 3-year EFS (75.281 ± 11.491% and 62.500 ± 21.348%) than those with CNS1 and TLP (97.998 ± 1.403% and 100%, *p* < 0.05).

## Discussion

The blood-brain barrier prevents most chemotherapeutic drugs from reaching effective concentrations in the CSF, making CNSL treatment a challenging endeavor. Nonetheless, the CNS is generally considered an ideal refuge for leukemia cells. Thus, early diagnosis and effective treatment are critical for CNSL patients.

Routine cytological examination of CSF is the gold standard for diagnosing CNS involvement in ALL. However, due to the small number of cells in CSF samples, it is challenging to distinguish primitive cells from normal lymphocytes only by morphology. Moreover, CM could be easily affected by subjective factors, with studies showing high false negative rate in morphological experiments, regardless of the modification of the Wright-Giemsa counterstaining method or CSF centrifugation [9, 10]. Therefore, using CM as a diagnostic criterion for CNSL may underestimate the morbidity of children with newly diagnosed ALL [4]. In our study, only 2.42% (4 out of 165) of newly diagnosed CSF specimens were CSF CM positive, compared with 20% for FCM, indicating the limitations of CSF CM. Moreover, 50% of the relapsed children had *BCR::ABL1* or *TCF3:: PBX1* fusion genes as a high-risk factor for CNS leukemia relapse, consistent with literature reports [11, 12]. However, in our study, no association was found between the CNS status and the frequency of fusion genes, including *ETV6::RUNX1*, *MLL-r*, *BCR::ABL1*, and *TCF3::PBX1*.

FCM is a comprehensive detection technology for rapid screening and quantitative analysis of single-row cells and biological particles in rapid linear motion. This technique can analyze tens of thousands of cells at high speed and measure multiple parameters from a single cell simultaneously. Moreover, FCM has several advantages over CM, including the ability to analyze a larger amount of data, higher sensitivity, multi-parameter analysis, faster processing, greater precision, and improved accuracy. FCM sensitivity can reach 10^− 4^, which is significantly higher than that of the CM method in CSF detection [9, 10]. This is because FCM uses the principle of antigen-antibody reaction to label each cell with the monoclonal antibody to distinguish the type of different cells and their characteristics, which is less affected by the cellular morphology. FCM also has a very thin “channel” that allows for each cell to pass through it individually. As each cell carries different immune markers on its surface, when a single cell passes through the channel, it will only react with the corresponding antigen and antibody, resulting in nearly 100% specificity.

Although the CM of CSF is considered to be a gold standard in the study of myeloid and lymphoid hematological tumors, its sensitivity is low, with false negative results ranging from 20 to 60% [10, 13], especially in low cell concentration samples, delaying the diagnosis and treatment of patients. Our study showed that the positive rate of leukemic cells detected by FCM was much higher than that of CM, and that FCM is more sensitive than CM when CSF contains only a small number of cells, which was consistent with the literature reports [5, 9, 14]. Furthermore, Ming Li [15] et al. found that the expression of the FLT3 gene in CSF can indicate that CNSL had occurred or is likely to occur. This suggests that the dynamic monitoring of specific gene expression in patients with acute leukemia using PCR technology could be used as a indicator for the diagnosis and monitoring of CNSL. Additionally, detecting circulating free DNA (cfDNA) in the cerebrospinal fluid is also of great significance in the diagnosis of CNSL and the study of the mechanism of its recurrence [12]. The application of CSF cfDNA sequencing in children with leukemia in the future may help to improve the detection rate of newly diagnosed CNSL, and to explore the mechanism of its recurrence.

Nevertheless, the leukemia cells are only found in the subarachnoid space; for patients with leukemic parenchymal infiltrates, both FCM and CM findings may be false-negative [16]. Our data also showed that in two patients with leukemic parenchymal infiltrates on enhanced magnetic resonance imaging of the brain, both FCM and CM results of CSF were negative. We believe that cranial imaging, such as cranial magnetic resonance imaging, is also essential to the initial assessment of CNS leukemia infiltration.

The optimal management of patients with CNSL remains uncertain. Up to now, the classic CNS-directed treatment for CNSL has been aslant from cranial radiation to high-dose methotrexate plus intrathecal chemotherapy. However, in the ALL-BFM95 trial, the BFM Collaboration found that adding two lumbar injections in induction therapy did not increase the EFS of CNS2 and TLP^+^ patients, and patients with CNSL at diagnosis had a higher risk of CNS relapses [17]. Furthermore, in the Dutch Childhood Leukemia Study Group (DCLSG) protocols ALL-7 and ALL-8 clinical trials, the Dutch Childhood Oncology Group collaboration found that patients with traumatic lumbar puncture had a worse prognosis (10-year EFS 58 + 7.6% versus 82 + 5.2%, *p* < 0.01), and no difference in outcome of patients with an initial CNS1 or CNS2 status and CNS2 patients did not receive extra intrathecal therapy in these studies [18]. Furthermore, Valérie’s research indicates that among patients in CNS2 status, those who test positive on flow cytometry may have a worse prognosis due to the potential for false-positive results in cytological examinations [6].

In our cohort of 165 childhood B-cell ALL patients, we found that 10 children with TLP^+^ had the same prognosis as those with TLP^−^ after 2 additional intrathecal injections during induction therapy, which may indicate this measure can improve the EFS of TLP^+^ patients. However, in our study, two additional intrathecal injections did not improve EFS in patients with CNS2 and CNS3, and CNSL at diagnosis was associated with a higher risk of CNSL relapse, consistent with the previous research finding [6]. These results may suggest that the presence of leukemia cells in the cerebrospinal fluid caused by traumatic lumbar puncture is distinct from true central leukemia at diagnosis. Additionally, intrathecal administration of chemotherapeutic agents can only reach the superficial layers of the brain. Therefore, the increased number of lumbar punctures may not have the same significant impact on patients with CNS2 and CNS3. Further research on the mechanism of central leukemia is needed to develop more effective and targeted treatments [19].

As few patients with ALL benefit from CRT as a component of CNS-directed treatment for de novo disease [20], cranial irradiation was omitted for all patients in our protocol. Because limited drugs can be involved in IT chemotherapy, in addition to increasing the number of lumbar punctures to strengthen the treatment, targeted drugs that can cross the blood-brain barrier will be selected to improve the efficacy and reduce the possibility of recurrence in children with CNSL [21–23]. Moreover, chimeric antigen receptor T cell (CAR-T) immunotherapy is a very effective treatment for children with single/multiply relapsed or refractory CNSL [24–26].

There were some limitations to our study. First, the follow-up period in our study was short. A long-term follow-up study is needed to address the prognostic significance of CNSL at diagnosis. Second, for children with positive fusion genes, such as *BCR::ABL1*, *TCF3::PBX1*, and *MLL-r*, our study did not test the genes expression in cerebrospinal fluid. Therefore, further investigation should be performed to detect the relationship of gene expression and recurrence.

## Conclusions

To conclude, our findings showed that the CSF FCM is superior to the traditional CM in assessing CNS involvement in ALL patients at the initial diagnosis and can be used as an important supplement to traditional CM. However, both FCM and CM findings may be false-negative, cranial imaging is also essential to the initial assessment of CNS leukemia infiltration. Moreover, CSNL at diagnosis is associated with adverse relapse prognosis, while the prognosis of patients classified as CNS2 and CNS3 does not improved with 2 additional intrathecal injections. Understanding the underlying mechanisms of CNS leukemia is imperative for the development of more efficacious and targeted therapeutic strategies.

## Data Availability

The data that support the findings of this study are available on request from the corresponding author.

## References

[1] Frishman-Levy L, Izraeli S (2017). Advances in understanding the pathogenesis of CNS acute lymphoblastic leukaemia and potential for therapy. Br J Haematol.

[2] Hunger SP, Mullighan CG (2015). Acute lymphoblastic leukemia in children. N Engl J Med.

[3] Correia CE, Schaff LR, Grommes C. Central nervous system Lymphoma:Approach to diagnosis and Treatment.Cancer J. 2020.26(3):241–52. 10.1097/PPO.0000000000000449 32496457

[4] Thastrup M, Marquart HV, Schmiegelow K. Flow Cytometric Detection of Malignant blasts in Cerebrospinal Fluid: a biomarker of Central Nervous System involvement in Childhood Acute Lymphoblastic Leukemia. Biomolecules. 2022;12(6). 10.3390/biom12060813 PMC9221543 35740938

[5] Dass J, Dayama A, Mishra PC, Mahapatra M, Seth T, Tyagi S (2017). Higher rate of central nervous system involvement by flow cytometry than morphology in acute lymphoblastic leukemia. Int J Lab Hematol.

[6] de Haas V, Pieters R, van der Sluijs-Gelling AJ, Zwaan CM, de Groot-Kruseman HA, Sonneveld E (2021). Flowcytometric evaluation of cerebrospinal fluid in childhood ALL identifies CNS involvement better then conventional cytomorphology. Leukemia.

[7] Arber DA, Orazi A, Hasserjian R, Thiele J, Borowitz MJ, Le Beau MM (2016). The 2016 revision to the World Health Organization classification of myeloid neoplasms and acute leukemia. Blood.

[8] Cai J, Yu J, Zhu X, Hu S, Zhu Y, Jiang H (2019). Treatment abandonment in childhood acute lymphoblastic leukaemia in China: a retrospective cohort study of the Chinese children’s Cancer Group. Arch Dis Child.

[9] Popov A, Henze G, Verzhbitskaya T, Roumiantseva J, Lagoyko S, Khlebnikova O (2019). Absolute count of leukemic blasts in cerebrospinal fluid as detected by flow cytometry is a relevant prognostic factor in children with acute lymphoblastic leukemia. J Cancer Res Clin Oncol.

[10] Ranta S, Nilsson F, Harila-Saari A, Saft L, Tani E, Soderhall S (2015). Detection of central nervous system involvement in childhood acute lymphoblastic leukemia by cytomorphology and flow cytometry of the cerebrospinal fluid. Pediatr Blood Cancer.

[11] Jeha S, Pei D, Raimondi SC, Onciu M, Campana D, Cheng C (2009). Increased risk for CNS relapse in pre-B cell leukemia with the t(1;19)/TCF3-PBX1. Leukemia.

[12] Sanchez R, Ayala R, Alonso RA, Martinez MP, Ribera J, Garcia O (2017). Clinical characteristics of patients with central nervous system relapse in BCR-ABL1-positive acute lymphoblastic leukemia: the importance of characterizing ABL1 mutations in cerebrospinal fluid. Ann Hematol.

[13] Bento LC, Correia RP, Alexandre AM, Nosawa ST, Pedro EC, Vaz ADC (2018). Detection of Central Nervous System Infiltration by myeloid and lymphoid hematologic neoplasms using Flow Cytometry Analysis: diagnostic accuracy study. Front Med (Lausanne).

[14] Del Principe MI, Buccisano F, Cefalo M, Maurillo L, Di Caprio L, Di Piazza F (2014). High sensitivity of flow cytometry improves detection of occult leptomeningeal disease in acute lymphoblastic leukemia and lymphoblastic lymphoma. Ann Hematol.

[15] Li M, Yang X, Jitao A, Chun X, Yuhua L, Ruomei L (2019). The expression and clinical significance of FLT3 gene in central nervous system leukemia. Jounal Practical Med.

[16] Crespo-Solis E, Lopez-Karpovitch X, Higuera J, Vega-Ramos B (2012). Diagnosis of acute leukemia in cerebrospinal fluid (CSF-acute leukemia). Curr Oncol Rep.

[17] Burger B, Zimmermann M, Mann G, Kuhl J, Loning L, Riehm H (2003). Diagnostic cerebrospinal fluid examination in children with acute lymphoblastic leukemia: significance of low leukocyte counts with blasts or traumatic lumbar puncture. J Clin Oncol.

[18] Dutch Childhood Oncology G, te Loo DM, Kamps WA, van der Does A, van Wering ER, de Graaf SS (2006). Prognostic significance of blasts in the cerebrospinal fluid without pleiocytosis or a traumatic lumbar puncture in children with acute lymphoblastic leukemia: experience of the Dutch childhood Oncology Group. J Clin Oncol.

[19] Zhou F, Wen Y, Jin R, Chen H (2019). New attempts for central nervous infiltration of pediatric acute lymphoblastic leukemia. Cancer Metastasis Rev.

[20] McNeer JL, Schmiegelow K (2022). Management of CNS disease in Pediatric Acute Lymphoblastic Leukemia. Curr Hematol Malig Rep.

[21] Ceppi F, Weitzman S, Woessmann W, Davies K, Lassaletta A, Reismuller B (2016). Safety and efficacy of intrathecal rituximab in children with B cell lymphoid CD20 + malignancies: an international retrospective study. Am J Hematol.

[22] Masuda K, Nakazato T, Nishiyama-Fujita Y, Ito C, Ogura S, Mizuno K (2019). Successful treatment with ponatinib for central nervous system relapse of Philadelphia chromosome-positive B-cell acute lymphoblastic leukaemia. Intern Med J.

[23] Shen S, Chen X, Cai J, Yu J, Gao J, Hu S (2020). Effect of Dasatinib vs Imatinib in the Treatment of Pediatric Philadelphia Chromosome-Positive Acute Lymphoblastic Leukemia: a Randomized Clinical Trial. JAMA Oncol.

[24] Tan Y, Pan J, Deng B, Ling Z, Song W, Xu J (2021). Toxicity and effectiveness of CD19 CAR T therapy in children with high-burden central nervous system refractory B-ALL. Cancer Immunol Immunother.

[25] Rubinstein JD, Krupski C, Nelson AS, O’Brien MM, Davies SM, Phillips CL (2020). Chimeric Antigen Receptor T Cell Therapy in patients with Multiply relapsed or refractory Extramedullary Leukemia. Biol Blood Marrow Transpl.

[26] He X, Xiao X, Li Q, Jiang Y, Cao Y, Sun R (2019). Anti-CD19 CAR-T as a feasible and safe treatment against central nervous system leukemia after intrathecal chemotherapy in adults with relapsed or refractory B-ALL. Leukemia.

